# An evaluation of Wb123 antibody elisa in individuals treated with ivermectin and albendazole, and implementation challenges in Africa

**DOI:** 10.11604/pamj.2017.27.65.11004

**Published:** 2017-05-29

**Authors:** Dziedzom Komi de Souza, Irene Offei Owusu, Joseph Otchere, Michelle Adimazoya, Kwadwo Frempong, Collins Stephen Ahorlu, Daniel Adjei Boakye, Michael David Wilson

**Affiliations:** 1 Department of Parasitology, Noguchi Memorial Institute for Medical Research, University of Ghana, Ghana; 2 Department of Epidemiology, Noguchi Memorial Institute for Medical Research, University of Ghana, Ghana

**Keywords:** Lymphatic filariasis, elephantiasis, antigen, antibody, Wb123, Ghana

## Abstract

The development of antibody testing for the diagnosis of lymphatic filariasis (LF) is intended to enhance the monitoring and evaluation activities of the Global Program for the Elimination of LF. This is due to the fact that antibody tests are expected to be the most sensitive at detecting exposure to LF compared to antigen that takes longer to develop. To this end a new antibody-based enzyme linked immunosorbent assay (ELISA) to *Wuchereria bancrofti* antigen Wb123 has been developed and further designed into a point of care rapid diagnostic test, under evaluation. In pre-treatment surveys, individuals were tested for antigen using the immuno-chromatographic test (ICT) card, and night blood microfilariae, after which all positives were treated using Ivermectin and Albendazole. The Wb123 ELISA was tested in antigen positive individuals, three months after they were treated. Samples were also tested for ICT and night blood microfilariae. The results revealed a reduction in microfilariae and ICT prevalence after treatment. Antigen and antibody prevalence increased with age. However, there was no correlation with the antibody responses observed. The mean WB123 antibody titers were higher among ICT positives, but not significantly different from ICT negative persons. While the Wb123 is targeted for use in untreated populations, further evaluations and guidelines will be required to define its use in populations that have undergone treatment for the control of LF.

## Introduction

Lymphatic filariasis (LF) is a neglected tropical disease (NTD) caused by infection with the parasitic worms *Wuchereria bancrofti*, Brugia malayi and B. timori [1]. The Global Programme to Eliminate Lymphatic Filariasis (GPELF) was launched in 2000 with the goal to eliminate LF by interrupting transmission through mass drug administration (MDA) and reducing morbidity and disability [2]. The adopted MDA strategy is annual treatment with a single dose of Albendazole in combination with either Ivermectin or Diethylcarbamazine (DEC) for 4-6 years [2]. The GPELF has achieved great success since its inception, providing a cumulative total of 5.62 billion treatments delivered to over 1 billion people at least once [3]. It is expected that by the targeted elimination goal of 2020, all endemic countries will have been verified as free of transmission or will have entered post-intervention (MDA) surveillance phase [1]. However, there are challenges to the program, one of which is a reliable tool to determine when it is appropriate to stop MDA and proceed with post-intervention surveillance [4]. This is especially due to the significant reduction in microfilaremia and antigenemia in endemic communities under treatment. The WHO recommends Transmission Assessment Surveys (TAS) for post-MDA surveillance [5] based on detecting circulating filarial antigen. This has a limitation of detecting infections only after the development of adult parasites. In view of this new surveillance tools are required. To this end, the development of antibody based assays targeting the third stage larvae has been proposed as an alternative, given the relatively faster appearance of antibodies in the blood, compared to antigens [6]. Various antibody based assays to recombinant filarial antigens have been developed, with challenges of cross-reactivity to other filarial parasites [6, 7]. These problems of specificity have however been addressed through the development of a new antibody based assay to *Wuchereria bancrofti* antigen Wb123 [8, 9]. While this assay is still under evaluation, we tested it in two communities with at least 12 rounds of yearly MDA.

## Methods

**Study sites**: Two LF endemic communities (Akonu and Agona Princess) in the Western Region of Ghana were selected for the study. Prior to MDA, areas in the region were identified as LF endemic with overall mf prevalence of 9-25% [10]. Both communities are rural. MDA with Albendazole and Ivermectin commenced in 2000.

**Ethics approval and consent to participate**: Approval for this study was obtained from the Ethical Review Board of the Noguchi Memorial Institute for Medical Research (IRB 077/13-14). Permission was sought from the chiefs and elders of the communities. Written informed consent was obtained from all eligible study participants.

**Sample collection and processing**: A cross-sectional survey was conducted in the communities in December 2014, 6 months after the MDA in June. Study participants above the gage of 5 years were examined for circulating filarial antigen (CFA), using the rapid immuno-chromatographic test cards (ICT cards Binax Now^®^, Inverness Medical Innovations Inc., USA BinaxNow) and microfilaria (mf). Microfilaria examination, using night blood collection, was done on only ICT positive individuals. Participants were screened for CFA using ICT cards, following the manufacturer's protocol. The giemsa stained thick blood smear (TS) and the Sedgwick-Rafter counting chamber (CC) methods were used for the quantification of microfilariae. One thousand microlitres (1 ml) of venous blood was drawn from each CFA positive individual between 21.00 and 24.00 hours into blood collection tubes containing Ethylenediamine Tetraacetic Acid (EDTA). 100 μl of blood was diluted in 900 μl of 3% acetic acid. The blood was transferred to a Sedgewick-Rafter counting chamber and examined for mf load, expressed as mf/ml of blood. For the thick smear method, 60 μl of blood was streaked onto a glass slide in three smears (20 μl each), stained with Giemsa and examined for mf. All ICT positive individuals were treated with Ivermectin (400 μg/kg) + 400 mg Albendazole, and subsequently retested 3 months after treatment. However, for the follow-up survey, the participants were also tested using the Wb123 enzyme linked immunosorbent assay (ELISA) (InBios). Five milliliters of venous blood was collected, from each study participant, in EDTA tube. After centrifugation at 6000 rpm for 5 min, plasma was collected and stored at -20°C for serological analyses. 10μl of plasma was diluted in 250μl of sample dilution buffer. 100μl of the dilution was used for the Wb123 Elisa, following the manufacturer's protocol [11]. Samples were run in two duplicates and the mean optical density (OD) normalized to the reference control provided with the Kit.

**Data analysis**: The parasitological indices determined included CFA prevalence, microfilaria prevalence and Wb123 prevalence. Prevalence rates were compared using a chi square test. All statistical tests were conducted at 95% confidence interval.

## Results

**Figure 1:**
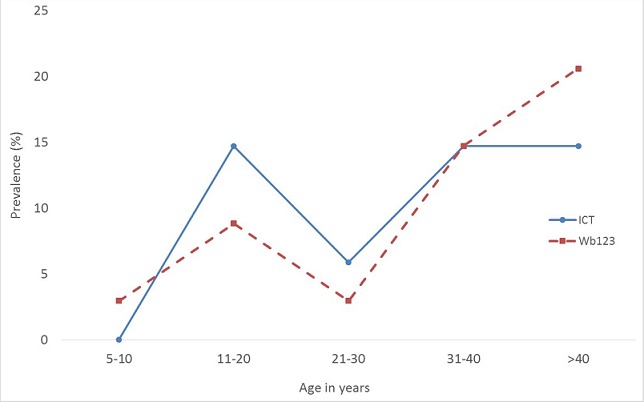
Age-specific prevalence of antigenemia and antibody responsiveness

370 individuals were recruited for the study. Of these, 48 (13.0%) were positive for ICT and 17 (4.4%) positive for mf. Three months after treatment, 34 previously positive individuals were tested, out of which 17 (50%) were positive for ICT and 17 (50%) for Wb123 (see Supplementary Material 1). Only one person (above 80 years) was positive for mf. This individual was also positive for both ICT and Wb123. The mean antibody titers were higher among ICT positives, but not significantly different from ICT negative persons (P>0.05). In this group of study participants, there was only one person aged 10 years. This individual was antibody positive but antigen negative. Six other individuals aged between 14 and 57 were also antibody positive but antigen negative. On the other hand, eight individuals aged between 12 and 71 were antigen positive but antibody negative. The age-specific prevalence of antigenemia and antibody responsiveness are shown in Figure 1, and reveal that both antigen and antibody prevalence increased with age. Pair-wise comparisons of test results were considered as the pair-wise sensitivity of the tests. Wb123 assay picked up 53% of the ICT positive results, whereas ICT picked up 56% of the Wb123 positive results. Negative test concordance revealed that 59% of ICT negatives were negative for Wb123, while 56% of Wb123 negatives were negative for ICT Table 1.

**Table 1:** Positive and negative concordance between tests.

Positive to positive concordance			Negative to negative concordance		
	Wb123	ICT		Wb123	ICT
Wb123		9/17 (53%)	Wb123		10/17 (59%)
ICT	9/16 (56%)		ICT	10/18 (56%)	

## Discussion

In this study, the observed reduction in mf prevalence after treatment indicates the effectiveness of the drugs in clearing microfilariae in the blood [12]. However, the lingering presence of antigen in microfilaria negative individuals may represent residual adult worm antigens from resolved infections that may persist for a couple of years [13]. While the presence of Wb123 reflects exposure to infective parasite, the implications of our observations to LF programs are not clear. On one hand, antibody responses develop before patent infection [14] and as such, positive responses may be suggestive of recent filarial exposure. On the other hand, antibodies take a long time (sometimes years) to normalize after treatment [9], and detection of antibody response in populations that have been treated only shows that these have been exposed at some point. This represents significant data interpretations challenges for the Wb123 assay.

Given the sample size evaluated in our study, a much bigger population will be required to further ascertain this observation. There are also some limitations to the current study that will require further evaluations. First, the Wb123 assay has not been validated for plasma samples. Secondly, the assay was not tested in the pre-treatment samples. There was not enough blood samples left, for the test to be undertaken. Secondly, the assay was undertaken on samples collected only three months after treatment and there will be the need to further evaluate the antibody responses, six months to a year after treatment. This will enable a better interpretation of the results, and definition of threshold prevalence levels. In undertaking this assay, the manufacturer recommends the end users to calculate cut-off values first using geographically relevant specimens. For this, “a minimum of 100 specimens are recommended for determination of the appropriate cut-off” [11]. This however could not be followed. Most country programs are currently at a stage where LF positivity in endemic areas are very low and thousands of individuals will have to be screened, before the minimum of 100 specimen for diseased individuals could be obtained. This requirement therefore presupposes that countries should obtain these specimen and determine cut-offs, before implementing the assays. In view of this we recommend the creation of a database for Wb123 assay results. Based on this, regional cut-offs could be determined for use by LF control programs.

## Conclusion

The Wb123 assay may be a good tool for assessing infection in populations that have not been treated, such as in children born after the implementation of MDA [8]. However, the estimates of antibody and antigen prevalence observed in this study do not correspond in a predictable manner. As such, using Wb123 assay in areas undergoing MDA or post-intervention settings will need to be given careful thought, taking into consideration the target population. There will be the need for guidelines defining the conditions under which the Wb123 assay can be used, as well as the interpretation of the results.

### What is known about this topic

- New tools are needed for post-MDA evaluation of LF in disease endemic settings;
- The Wb123 assay represents a new generation tool with the possibility of predicting on-going transmission.

### What this study adds

- The use of the Wb123 assay requires the prior determination of appropriate cut-off points, using known samples. This will be a challenge towards the end of control programs, with a difficulty in obtaining positive samples;
- Guidelines for data interpretation will also be required for implementation

## Competing interests

The authors declare that they no competing interests.
